# Force Variability during Dexterous Manipulation in Individuals with Mild to Moderate Parkinson’s Disease

**DOI:** 10.3389/fnagi.2015.00151

**Published:** 2015-08-10

**Authors:** Na-hyeon Ko, Christopher M. Laine, Beth E. Fisher, Francisco J. Valero-Cuevas

**Affiliations:** ^1^Brain-Body Dynamics Laboratory, Department of Biomechanical Engineering, Division of Biokinesiology and Physical Therapy, University of Southern California Los Angeles, Los Angeles, CA, USA; ^2^Neuroplasticity and Neuroimaging Laboratory, Division of Biokinesiology and Physical Therapy, University of Southern California Los Angeles, Los Angeles, CA, USA

**Keywords:** Parkinson’s disease, sensorimotor control, fingers, dexterity, dynamic force control, force variability, clinical evaluations

## Abstract

Parkinson’s disease (PD) is a progressive neurodegenerative disease affecting about 1–2% of the population over the age of 65. Individuals with PD experience gradual deterioration of dexterous manipulation for activities of daily living; however, current clinical evaluations are mostly subjective and do not quantify changes in dynamic control of fingertip force that is critical for manual dexterity. Thus, there is a need to develop clinical measures to quantify those changes with aging and disease progression. We investigated the dynamic control of fingertip forces in both hands of 20 individuals with PD (69.0 ± 7.4 years) using the Strength–Dexterity test. The test requires low forces (<3 N) to compress a compliant and slender spring prone to buckling. A maximal level of sustained compression is informative of the greatest instability the person can control, and thus is indicative of the integrity of the neuromuscular system for dexterous manipulation. Miniature sensors recorded fingertip force (F) during maximal sustained compressions. The force variability during sustained compression was quantified in two frequency bands: low (<4 Hz, F_LF) and high (4–12 Hz, F_HF). F_LF characterizes variability in voluntary fluctuations, while F_HF characterizes variability in involuntary fluctuations including tremor. The more-affected hand exhibited significantly lower F and lower F_LF than those in the less-affected hand. The more-affected hand showed significant negative correlations between F_LF and the Unified Parkinson’s Disease Rating Scale motor scores for both total and hand-only, suggesting that greater force variability in the voluntary range was associated with less clinical motor impairment. We conclude the nature of force variability in the voluntary range during this dynamic and dexterous task may be a biomarker of greater motor capability/flexibility/adaptability in PD. This approach may provide a more quantitative clinical assessment of changes of sensorimotor control in individuals with PD.

## Introduction

Parkinson’s disease (PD) is the second most common neurodegenerative disease in the United States, affecting about 1–2% of the population over age of 65 (Guttmacher et al., 2003; de Lau and Breteler, 2006; Weintraub et al., 2008). Loss of hand dexterity and impaired sensorimotor control of grip force have been reported in PD (Gordon et al., 1997; Ingvarsson et al., 1997; Fellows et al., 1998; Gordon, 1998; Nowak and Hermsdörfer, 2006; Lawrence et al., 2014; Lukos et al., 2014). The gradual impairment of dexterous manipulation leads to difficulties in daily activities, such as buttoning, eating, extracting money from a wallet, or signing a check (Lukos et al., 2014). Loss of these abilities will negatively impact qualify of life (Lukos et al., 2014).

The Unified Parkinson’s Disease Rating Scale (UPDRS) is the most well-established and accepted assessment in PD (Ramaker et al., 2002; Goetz et al., 2008). The motor examination portion of the UPDRS (part III) provides a global motor severity score, but does not measure force control. The ability to dynamically regulate both the magnitude and direction of fingertip force vectors is fundamental for dexterous manipulation (Cole and Abbs, 1988; Valero-Cuevas et al., 1998, 2003), and can be revealing of sensorimotor processing capability in older adults (Dayanidhi and Valero-Cuevas, 2014; Lawrence et al., 2015). This ability progressively deteriorates with the progression of PD, but the physiology of this process is not well understood. Therefore, it is critical to develop a sensitive measure of the neural control of fingertip force vectors in PD. Such a measure would add an informative and currently missing component to the current set of clinical assessment tools used for PD.

In the past, quantification of dynamic dexterous manipulation ability in PD has been difficult because of lack of appropriate techniques (Lukos et al., 2014). The Strength–Dexterity test was developed to quantify dynamic dexterous manipulation in general, and has been used to measure finger dexterity in healthy individuals (4–89 years) and those suffering from pathological conditions, such as carpometacarpal osteoarthritis, PD, and children with pollicized hands (Vollmer et al., 2010; Dayanidhi et al., 2013; Dayanidhi and Valero-Cuevas, 2014; Lawrence et al., 2014; Lightdale-Miric et al., 2015). The previous study of dynamic dexterous manipulation in PD, however, did not consider different degrees of motor symptoms between the hands (Lawrence et al., 2014) despite the fact that lateralized motor impairment is common in PD (Lukos et al., 2014). Differences in dynamic force control between the more- and less-affected hands could be highly informative, given that motor symptoms likely affect dynamic dexterous manipulation.

Measures of dynamic force control during the Strength– Dexterity test might reveal sensorimotor impairment in PD. fMRI studies have shown that the basal ganglia are active during the sustained spring compressions of the Strength–Dexterity test (Mosier et al., 2011; Pavlova et al., 2015) (in press). In addition, the blood–oxygen-level dependent (BOLD) signals in the putamen increased as the spring became more unstable (Mosier et al., 2011). Given that disruption of the basal ganglia result in motor impairment in PD, and that the basal ganglia are known to be involved in the spring task, it is likely that measuring the dynamic control of fingertip forces during performance of the Strength–Dexterity test may provide a sensitive index of manual sensorimotor control in PD.

Therefore, the purpose of this study was to explore differences in dynamic control of fingertip forces between the more-affected and less-affected hands in individuals with PD. If such differences exist, it would indicate that measures of force during the spring task hold a potential as markers of symptom severity that may not be evident with traditional clinical testing. As a further evaluation of this potential, spring force measures were correlated with the well-established clinical assessment of motor impairment, the UPDRS. Thus, we respond to the goal of this Research Topic to develop clinical measures to enable future studies of the mechanisms of declining motor control in aging and disease.

## Materials and Methods

### Participants

A total of 20 individuals with PD (69.0 ± 7.4 years, 11 M, 9 F) participated in the study. Given the observational, cross-sectional nature of this study, we included patients with a diagnosis of PD who were functionally independent (regardless of their medication status) and demonstrated intact cognitive functions and excluded patients with musculoskeletal symptoms including pain and fatigue as well as a history of other neurological disorders and surgical procedures affecting the thumb and index finger. The study was approved by the Institutional Review Board at the University of Southern California. Informed consent was obtained from all participants. The average disease duration for 20 individuals with PD was 6.0 years (±4.1 years), and all participants were physically independent and Hoehn and Yahr stages 1–3. Eighteen participants were on PD medications while two participants did not take PD medications. We included participants both on- and off-medication because our study represents a cross-sectional and exploratory investigation of dynamic fingertip force control in the general population of functionally independent patients with PD. The more-affected side was determined by UPDRS motor examination and self-report from patients asked, “*which hand has been giving you more trouble in daily activities?*” UPDRS motor scores were only obtained from a subset of 13 patients. Thus, the more-affected side was self-reported from seven patients whose UPDRS scores were not available and also from two patients whose UPDRS scores were the same for both hands. Handedness was also measured by the Edinburgh Handedness Inventory at the screening. However, a subsequent multiple regression analysis revealed handedness did not influence dynamic fingertip force control. This is in line with findings reported for the healthy population (Lawrence et al., 2014).

### Instrumentation for dynamic fingertip force measurement

**Figure 1:**
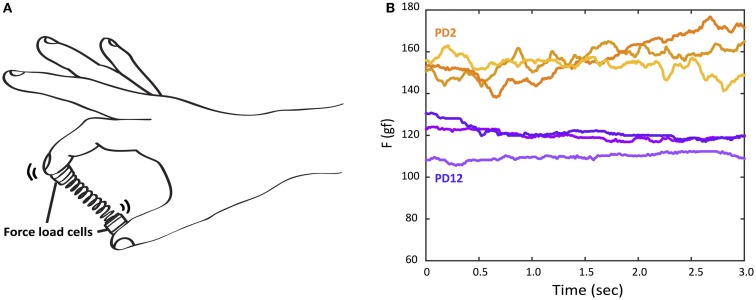
**The Strength–Dexterity test and raw force data examples of three trials from the more-affected hand of two PD participants with different UPDRS hand motor scores**. **(A)** Participants compress a slender spring prone to buckling as much as they can to its solid length, sustain the compression for 5 s, and release the compression. The force data were recorded from miniature load cells at the tips of spring. **(B)** *Orange lines:* the top three force traces during a hold phase from PD2 with UPDRS (more-affected) hand motor score, 7. *Purple lines:* the top three force traces during a hold phase from PD12 with UPDRS (more-affected) hand motor score, 15.

The Strength–Dexterity test was used to measure dynamic sensorimotor control of fingertip force from the thumb and index finger. The test required compressing a slender spring with the thumb and index fingers without allowing it to buckle (Figure 1) (Valero-Cuevas et al., 2003; Dayanidhi et al., 2013). The specifications of the custom spring (Century Springs Corp., Los Angeles, CA, USA) were the following: (1) free length = 3.96 cm, (2) solid length = 0.69 cm, (3) force range = 0–2.84 N, (4) stiffness = 0.86 N/cm, and (5) the diameter of end caps were 0.95 cm (Figure 1) (Dayanidhi et al., 2013). The spring was designed to be impossible to compress fully, and thus the maximal compression participants could achieve was less than 3N (Dayanidhi et al., 2013). As the spring is compressed, it becomes increasingly unstable in a non-linear way (Venkadesan et al., 2007), making it unpredictable and also making the particular dynamics of each sustained compression unique. The maximal level of compression that is sustained reflects the integrity of the sensorimotor system, which controls fingertip force and direction during object manipulation (Dayanidhi et al., 2013; Dayanidhi and Valero-Cuevas, 2014; Lawrence et al., 2014).

### Experimental procedure

The participants were seated comfortably with their forearm supported by a foam pad. They were asked to pick up the spring with the thumb and index finger and familiarize themselves with the properties of the spring. As in our prior studies, the number of trials was varied among patients as needed in order to make them familiar with the test and produce relatively consistent performance with each attempt. They were asked to either open or curl the other three fingers so as not to touch or assist the index finger. The instruction given to the participants was “*compress the spring as much as possible without buckling, hold the compression for 5 s, and release the compression*.” We measured both affected and less-affected hands. All participants were tested with their less-affected hand first to ensure that they fully understood the task before testing with the more-affected hand. Since the task requires dynamic control of an unpredictable object, it is unlikely that the testing order would influence performance. Only hold periods where force was held stable for at least 3 s were used for further analysis. The goal of the experiment was to obtain the highest compression force possible. The three trials with the highest compression forces (per hand) were used for further analysis. We chose to analyze only the three best trials per subject to minimize potential sources of variance related to learning, task-familiarization, and sub-maximal (overly cautious) efforts.

### Data collection and analysis

Customized miniature load cells (ELB4–10, Measurement Specialties, Hampton, VA, USA) at the end caps were used to measure fingertip force in the compression direction. The load cells were connected to a signal conditioner and USB-DAQ (National Instruments, Austin, TX, USA). The signals were sampled at 400 Hz with a custom-written MATLAB (MathWorks, Natick, MA, USA) program.

For a particular sustained compression period to be used in further analysis, the compression force was required to remain within 1 SD of mean force recorded during the attempt (Dayanidhi et al., 2013).

In addition to measuring the maximal mean sustained compression force for each trial, force variability was analyzed at two frequency bands to distinguish slow voluntary force fluctuations (<4 Hz) from fast involuntary force fluctuations (4–12 Hz) that include tremor, a well-known symptom in PD. The first was aimed at quantifying voluntary fluctuations in force, produced as the subjects attempted to control the buckling of the spring by dynamically altering the magnitude and direction of their fingertip forces. These voluntary fluctuations occur at low frequencies (<4 Hz) (Miall et al., 1993; Slifkin et al., 2000; Vaillancourt et al., 2001). We quantified them simply as the SD of the sustained compression force after applying a 4 Hz low-pass filter (zero-phase, fourth order Butterworth) to the force. SD is a commonly reported measure because of its simplicity, compatibility with prior literature on force control in PD (Slifkin and Newell, 1999; Vaillancourt et al., 2001, 2002), and lack of dependence on the duration of the hold period or the mean value of the signal. We did remove any linear trend for each sustained compression prior to calculation of SD to prevent it potential inflation by such slow trends. This measure of low-frequency force dynamics is referred to herein as F_LF.

The second measure of force dynamics was aimed at quantifying faster, involuntary fluctuations, which include tremor oscillations and noise from the motor system. For this analysis, the force signal during each sustained compression was band-pass filtered between 4 and 12 Hz (zero-phase, fourth order Butterworth) and the RMS of the resulting signal was calculated. The RMS of the band-pass filtered force trace gives a value, which is directly comparable and mathematically related to the signal power in the frequencies present. The most common way to quantify “tremor-band” activity is by a measure of spectral power (McAuley and Marsden, 2000; Vaillancourt et al., 2001), thus, our analysis is in keeping with standard methodology while taking advantage of the simplicity and robustness of time-domain calculations of signal variance. This measure of high frequency force dynamics is referred to herein as F_HF.

### Statistical analysis

To quantify differences in each measure of force between the more- and less-affected hands, we first checked all distributions for normality using a Lilliefors test. Force measures that showed non-normal and skewed distributions were normalized using a log transformation before testing for differences of means. To test for differences in means, we used a 10,000 iteration permutation test on paired-differences (Hooton, 1991; Ludbrook, 1994). This test directly determines the probability that the mean paired-difference between two data sets could have occurred by chance (i.e., after randomly changing the sign of each paired-difference). We chose this non-parametric test over a repeated measures ANOVA design for its robustness and lack of assumptions regarding the distribution of variances across subjects and trials. The method directly tests the null hypothesis that hand designation, such as more-affected vs. less-affected, had no effect on the force measurement.

Where differences were found between hands at the group level, we determined the directional consistency of the effect at the subject level by calculating an average difference in each force measure per subject. If significantly more than 50% of subjects showed a directional difference across hands according to a binomial test, we considered the effect to be generalizable at the subject level. If not, we can assume that a subset (e.g., those with more severe symptoms) were primarily responsible for the group effect.

Finally, we tested all force measures – and the magnitude of their differences across hands – for correlation with the UPDRS motor scores obtained for a subset of 13 participants out of the original 20. In particular, we tested for correlation to (i) the entire UPDRS motor score, (ii) the UPDRS hand-only score for the more-affected and the less-affected hands, and finally (iii) the UPDRS motor score excluding all hand scores (non-hand motor score). Given that UPDRS tremor scores have received recent attention as potentially descriptive for PD classification (Stebbins et al., 2013), we also tested our force measures for correlation with UPDRS hand-tremor scores. These were calculated as the sum of the postural tremor, kinetic tremor, and rest tremor amplitude evaluations within the UPDRS. To be conservative, we used the non-parametric Spearman’s rho rank correlation, with the significance of each coefficient determined by a permutation test. This test calculated the correlation between force measures and UPDRS scores before and after shuffling the UPDRS score assignments across subjects, replacing the scores from one subject with the scores from another. The probability that the correlation coefficient obtained could have occurred by chance was thus directly calculated from 10,000 sets of shuffled data. This permutation process allowed all 3 trial replicates for the 13 subjects to be used, rather than reducing the data set to 13 mean values. This allowed us to test for the significance of correlations in a conservative and assumption-free way.

In this study, we calculate a large number of correlations. Because each test is deemed significant at the 95% confidence level, we can expect that 5% of independent tests might show significance by chance. This is important if we interpret the occurrence of a single significant result to imply clinical utility for the Strength–Dexterity test. We do not specifically make this claim; nonetheless, we used a binomial test to determine if the number of significant correlations observed could have occurred by chance given the number of statistical tests. The approach directly addresses the problem of multiple comparisons without requiring the global adjustment of confidence levels.

## Results

### Force measures

We found significant group differences in dynamic fingertip force control between the more- and less-affected hands during the Strength–Dexterity test. The basic group-level differences in F, F_LF, and F_HF are as follows.

#### Mean Compression Force (F)

The mean compression force measured from the more-affected hand was significantly lower (i.e., worse) than that of the less-affected hand (*p* = 0.019). Interestingly, although the difference was significant at the group level, 60% of individual participants showed greater (i.e., better) F in the less-affected hand. For 20 participants, 60% is essentially chance-level according to a binomial test, thus, a difference between hands in compression force was not, on average, directionally consistent across PD patients.

#### SD of Force Fluctuations <4 Hz (F_LF)

The mean of F_LF was significantly lower in the more-affected hand (*p* = 0.042) than in the less-affected hand at the group level. Only 50% of tested individuals displayed greater mean F_LF in the less-affected hand than in the more-affected hand, indicating a subgroup-driven effect rather than a general feature of PD.

#### Root Mean Square of Force at 4–12 Hz (F_HF)

No significant mean difference was found for F_HF between hands.

The heterogeneity of symptom severity among individuals may have influenced these results and is further explored below at the individual level.

### UPDRS motor scores and force measures

The MDS-UPDRS (the revised version by the movement disorder society) motor examination scores were obtained from 13 of the 20 participants by a trained and certified clinician. Twelve participants were on medication while one participant (PD1) in the early stage of disease voluntarily delayed drug therapy. The total motor scores ranged from 7 to 53 among the 13 participants (Table 1). The lower the UPDRS motor scores, the lesser the motor impairment.

**Table 1:** **Clinical characteristics of 13 patients with Parkinson’s disease**.

PD no.	Age	Sex	Disease duration (years)	Affected hand	H and Y stage	UPDRS motor score			Medication
						Total	More-affected hand	Less-affected hand	
1	70	F	2	R	1	32	12	6	Off
2	70	M	0.4	R	1	7	7	0	On
3	55	F	3	R	1	32	14	6	On
4	66	M	0.33	L	2	26	11	7	On
5	73	F	7	L	2	17	4	3	On
6	76	F	8.75	R	2	27	8	8	On
7	65	F	8	L	2	22	7	5	On
8	72	F	3.75	R	2	53	17	12	On
9	71	M	3	L	2	41	12	9	On
10	68	M	4	R	2	34	9	6	On
11	71	M	4	L	3	52	12	12	On
12	80	M	2.5	R	2	43	15	8	On
13	75	F	7	R	2	28	12	9	On

#### Correlations Between the UPDRS Total Motor Score and Force Measures (F, F_LF, and F_HF)

Table 2 summarizes Spearman’s rho rank correlation coefficients and *p*-values between the UPDRS total motor scores and force measures between two hands at the group level. Only F_LF showed a significant correlation in the more-affected hand (ρ = −0.44, *p* = 0.04).

**Table 2:** **Spearman’s rho coefficients (ρ) and *p*-values between UPDRS motor scores and force measures**.

UPDRS	More-affected hand						Less-affected hand					
	Total motor		Hand only		Non-hand		Total motor		Hand only		Non-hand	
	ρ	*p*-value	ρ	*p*-value	ρ	*p*-value	ρ	*p*-value	ρ	*p*-value	ρ	*p*-value
F	−0.11	0.35	−0.22	0.23	−0.20	0.25	−0.006	0.49	0.096	0.37	−0.16	0.29
F_LF	−0.44	0.04*	−0.52	0.016*	−0.39	0.062	−0.024	0.46	−0.16	0.24	0.05	0.43
F_HF	−0.26	0.16	−0.14	0.30	−0.42	0.060	0.18	0.25	0.067	0.41	0.16	0.28

*Only low-frequency force fluctuation was significantly correlated with the UPDRS total motor and hand-only motor scores*.

*p < 0.05, statistical significance was determined by a 10,000 iteration permutation test.

#### Correlations Between the UPDRS Hand-Only Motor Score and Force Measures

**Figure 2:**
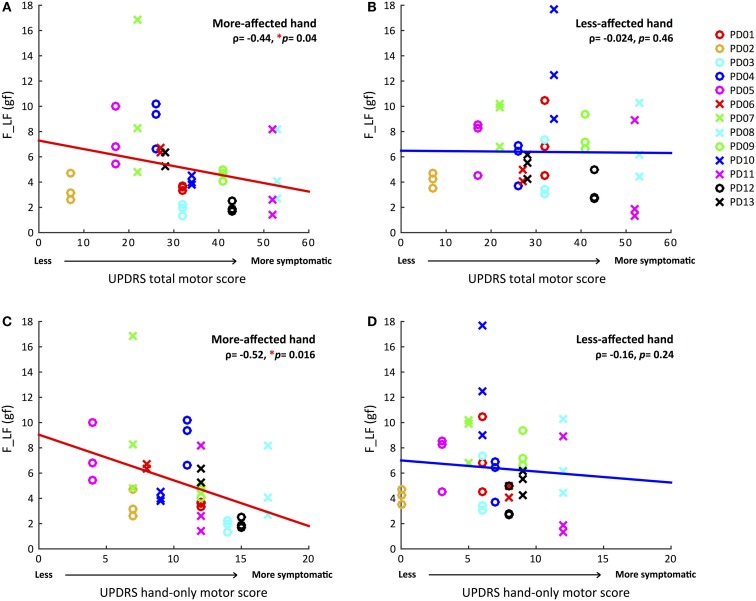
**Correlations between magnitudes of voluntary force fluctuations and UPDRS total and hand-only motor scores for both hands**. **(A)** Greater voluntary force fluctuations correlated with less total motor impairment in the more-affected hand. **(B)** No significant correlation between voluntary force fluctuations and UPDRS total motor score in the less-affected hand. **(C)** Greater voluntary force fluctuations associated with less hand-related motor impairment in the more-affected hand. **(D)** No significant correlation between voluntary force fluctuations and UPDRS hand-only motor score in the less-affected hand. (*p < 0.05, Statistical significance of each Spearman’s coefficient was determined by a 10,000 iteration permutation test. The linear fit was only for visual representations.)

For the UPDRS hand-only motor score, we considered a set of seven hand-related items from the full assessment list: rigidity, finger tapping, hand movements, pronation/supination, postural tremor, kinetic tremor, and resting tremor amplitude. The UPDRS hand-only motor score for the more-affected hands ranged from 4 to 17, and from 0 to 12 for the less-affected hand. Once again, only F_LF (ρ = −0.52, *p* = 0.016) showed a significant correlation in the more-affected hand (Figure 2). That is, greater variability of voluntary force fluctuations was associated with less motor impairment measured by UPDRS total and hand-only motor scores.

#### Correlations Between the UPDRS Non-Hand Motor Score and Force Measures

To quantify the general, non-hand related, motor impairment, such as gait and balance, the hand-only motor score for both hands was subtracted from the UPDRS total motor score. The UPDRS motor score without the hand scores negatively correlated with both F_LF (ρ = −0.39, *p* = 0.062) and F_HF (ρ = −0.42, *p* = 0.06) for the more-affected hand, although these correlations fell just short of statistical significance. Interestingly, these correlations were not found in the less-affected hand (Table 2).

#### Correlations Between the UPDRS Tremor Score and Force Measures

We derived a tremor score per each hand by summing scores from three UPDRS tremor-related items: postural tremor, kinetic tremor, and rest tremor amplitude. The tremor scores ranged from 1 to 7 for the more-affected hand, and 0 to 4 for the less-affected hand. We found no significant correlations between tremor scores and any of our force measures.

#### Correlations Between the UPDRS Total Motor Score and Between-Hand Difference in F, F_LF, and F_HF

**Figure 3:**
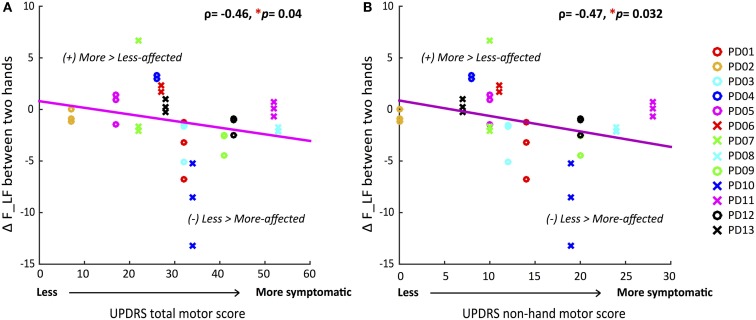
**Correlations between differences in voluntary force fluctuations between the more-affected and less-affected hands and UPDRS motor scores**. **(A)** Decrease of between-hand difference in AF_LF was significantly correlated with greater total motor impairment. **(B)** Decrease of between-hand difference in AFLF was significantly correlated with greater non-hand motor impairment. (*p < 0.05, statistical significance of each Spearman’s coefficient was determined by a 10,000 iteration permutation test. The linear fit was only for visual representations.)

Because only a subset of all participants influenced the group differences in F and F_LF (60 and 50%, respectively), due to heterogeneity of symptom severity among participants, we tested if the between-hand differences in force and force variability for each individual were correlated with overall motor impairment level. Table 3 summarized Spearman’s rho rank correlation coefficients and *p*-values between the UPDRS motor scores and between-hand difference in force measures at the individual level. The between-hand differences in force measures were calculated as the more-affected minus the less-affected hand in magnitude. A significantly negative correlation was again found only between the overall UPDRS motor score and ΔF_LF (ρ = −0.46, *p* = 0.039) (Figure 3). Less difference in F_LF between hands was associated with increased overall motor impairment.

**Table 3:** **Spearman’s rho coefficients (ρ) and *p*-values between UPDRS motor scores and between-hand difference in force measures**.

UPDRS	Total motor		Non-hand motor	
	ρ	*p*-Value	ρ	*p*-Value
ΔF	−0.083	0.38	−0.009	0.49
ΔF_LF	−0.46	0.04*	−0.47	0.032*
ΔF_HF	−0.39	0.076	−0.48	0.039*

*p < 0.05, statistical significance was determined by a 10,000 iteration permutation test.

#### Correlations Between the UPDRS Non-Hand Motor Score and Between-Hand Difference in F, F_LF, and F_HF

We determined if the magnitude of difference in force measures between the two hands were correlated with the more systemic and non-hand-related motor symptoms covered by the UPDRS motor examination. A significantly negative correlation was found for ΔF_LF (ρ = −0.47, *p* = 0.032) (Figure 3) once again, indicating that decrease in differences between hands corresponded to greater non-hand motor symptom severity, such as impairment of balance and gait. But additionally, ΔF_HF now showed a significant negative correlation (ρ = −0.48, *p* = 0.039) (Table 3) with UPDRS, indicating that larger differences between hands in involuntary force fluctuations corresponded to less systemic motor impairment.

Because a large number of correlations were tested for statistical significance, we used a binomial test (Dodge, 2008) to determine if the overall proportion of correlations exceeding the 95% confidence level was greater than would be expected given the number of tests executed. Many of our tests are not independent, however, to be extremely conservative, we assumed 24 independent tests (every test in Tables 2 and 3). The binomial probability that we would have obtained five significant results by chance is *p* = 0.00019. Of course, reducing the number of independent tests can only strengthen our results.

## Discussions and Conclusion

Measures of dynamic force control during the Strength–Dexterity test, an inherently dynamical and dexterous task, revealed characteristic differences between the more- and less-affected hands in PD, an aging population with progressively declining hand function. The purpose of the paper was to explore force control strategies during a dynamic and dexterous task. Measurements of dynamic finger force may begin to fill the need for more objective and sensitive measures of sensorimotor function to better chart the progression of disease and gage treatment. Note: although we speak of maximal sustained compression forces and variability therein, these maximal forces are all <3 N (<10% maximal static pinch force). The Strength–Dexterity test is predicated on the notion that studying precision manipulation with the fingertips at low force magnitudes while pushing the motor system to a limit of dynamical performance (i.e., the edge of instability) is informative of the integrity and deficits in the neuromuscular mechanisms for sensorimotor control in manipulation (Venkadesan et al., 2007; Dayanidhi et al., 2013; Dayanidhi and Valero-Cuevas, 2014; Lawrence et al., 2014, 2015; Duff et al., 2015).

The main finding of the study concerns the force fluctuations at low frequencies (in the voluntary range <4 Hz, F_LF) seen during the maximal level of sustained compression. We found lower variability at these frequencies was associated with greater severity of motor impairment measured by the UPDRS total and hand-only motor scores. Thus, measures of force variability during the performance of the Strength–Dexterity test hold potential as objective clinical assessment tool in PD, and may be a useful addition to current clinical assessments for characterizing and tracking the severity of both hand and general motor impairment.

Many individuals with PD naturally show greater motor impairment in one hand compared with the other (Jankovic, 2008; Lukos et al., 2014). Because of this, we sought to identify group differences in dynamic force control between the more- and less-affected hands. We found that the more-affected hand compressed the unstable spring with less force and with reduced low-frequency force fluctuations (<4 Hz) compared with the less-affected hand. Slow fluctuations in force relate mostly to active and voluntary strategies and adjustments to stabilize the unstable object. Since the instability of the spring increases with compression force, our finding of decreased compression force in the more-affected hand implies reduced ability to control instability (Venkadesan et al., 2007). This reduced control of instability appears to influence both compression force and force variability. However, our data suggest that compression force and low-frequency force variability may reflect relatively independent aspects of stability control in PD because a subsequent analysis showed no significant correlation between compression force and low-frequency force variability (more-affected side: ρ = 0.32, *p* = 0.11, less-affected side: ρ = 0.25, *p* = 0.2). Interestingly, force fluctuations at higher frequencies (4–12 Hz), which includes tremor (Vaillancourt et al., 2001; Jankovic, 2008), a well-known symptom in PD, were not different between the two hands. PD may also be classified into tremor dominant and postural instability/gait difficulty groups with UPDRS measures (Stebbins et al., 2013). Given the potential importance of tremor for disease categorization, we also explored the relationship between UPDRS tremors scores and force measures. We found no significant correlations indicating that our measures are not directly affected by tremor symptoms measured in the UPDRS. These findings suggest that force variability during the Strength–Dexterity test is most sensitive to impairment of voluntary rather than reflexive and involuntary aspects of sensorimotor control.

We examined if the force measures (F, F_LF, and F_HF) reflected hand-specific motor symptom severity. We found that the F_LF, low-frequency force fluctuations significantly negatively correlate with UPDRS measures only in the more-affected hand. This indicates that greater low-frequency fluctuations during the Strength–Dexterity task are associated with less impairment level of the more-affected hand. The same significant correlation was found for the UPDRS total motor score. The UPDRS non-hand motor score showed this same trend, albeit at a non-significant level.

The inevitable diversity of symptom severity in our participants may have affected our group comparisons. Therefore, we analyzed differences in force dynamics between hands. This within-subject analysis showed that it was mostly the participants with greater impairment that exhibited decreased F_LF in the more-affected hand relative to the less-affected hand. This was also the case for the UPDRS non-hand motor score. The latter finding is particularly interesting, because it suggests that ΔF_LF between hands may be indicative of systemic and general motor dysfunction.

Furthermore, the difference in high frequency force fluctuations (ΔF_HF) between hands correlated only with the UPDRS non-hand motor score. It may be that high frequency force fluctuations could reflect mostly systemic and general motor impairment. The magnitude of maximal sustained compression force, F, although different across hands on average, did not correlate well with any UPDRS measure. Thus, force fluctuations during the sustained compressions are likely more informative of neural control capabilities than the level of compression itself.

Given that low-frequency force fluctuations were smaller in hands with greater levels of motor impairment, it is reasonable to speculate that the reduced variability represents a loss of compensatory mechanisms employed by PD patients to control instabilities with the more-affected hand. Previous research showed greater variability in various force generation tasks in PD patients relative to controls (Sheridan et al., 1987; Stelmach et al., 1989; Vaillancourt et al., 2002). While increased force variability in PD might indicate impairment under some conditions, the within-subject design of the present study compels an alternative interpretation of force variability. In some contexts, motor variability may reflect flexibility or adaptability of motor systems (Vereijken, 2010). Variability in a physiological process is thought to be necessary to adapt to unpredictable environmental changes, and this capability decreases with aging (Lipsitz and Goldberger, 1992). In the present task of controlling an unstable compliant object, the correlation between increased clinical motor impairment and reduced force variability may represent a progressive failure of the PD motor system to employ flexible/adaptive strategies for stabilizing the spring. Thus, our findings have important consequences to our understanding of variability and motor impairment in PD because it shows that not all variability is detrimental. We suggest, therefore, that such changes in variability with disease progression during a highly dynamical and complex stabilization task (i.e., as the system is pushed to some limit of performance) are informative of motor impairment in PD.

It is also possible that individuals with PD employ a fundamentally different motor strategy when using their more-affected hand relative to their less-affected hand. The Strength–Dexterity task requires mainly online somatosensory feedback to control the unstable spring. It is reported that in general, individuals with PD rely more heavily on visual feedback to guide motor actions (Cooke et al., 1978; Gordon et al., 1997; Redgrave et al., 2010). We, however, have seen reliance on slower and less effective visuomotor corrections only when tactile sensation is removed in healthy individuals (Venkadesan et al., 2007). Greater reliance on visual feedback could enhance force variability (Shadmehr et al., 2010); however, the advantages and disadvantages of visual strategies in the context of our study are unknown. Thus, it could be that the reduced force variability in the more-affected hand reflects a compensatory adaptation to impaired tactile and proprioceptive control.

The reflexive/reactive/low-level component of dexterous manipulation, however, is relatively preserved in PD. Reactive force control by a perturbation during in-hand manipulation takes about 70 ms (Cole and Abbs, 1988; Johansson and Cole, 1992), and continuous updating of somatosensory information and motor response may even shorten to about 40–50 ms (Johansson et al., 1992). The PD motor system seems to preserve intact neural control for early reflexive responses to the perturbation (Ingvarsson et al., 1997; Fellows et al., 1998). Furthermore, the short latency reflex is intact in PD (Rothwell et al., 1983; Cody et al., 1986). In our study, high frequency force fluctuations, which may reflect this reflexive/reactive/low-level component of task performance, were not different between the more and less-affected hands. Only the difference in this measure between hands was significantly correlated with UPDRS non-hand motor score. This would seem to support the idea that PD influences the active/voluntary/high-level aspects of dexterous manipulation more so than reflexive/reactive/low-level of control aspects.

Interestingly, both ΔF_LF and ΔF_HF between the two hands showed a significant negative correlation with non-hand-related motor scores [i.e., systemic and gross motor function (Lawrence et al., 2014)]. These findings suggest that dynamic fingertip forces measured within the context of a voluntary task may still provide information about the degree of systemic motor impairments, such as alteration of posture, gait, and balance, suggesting some commonality of neural circuitry in the system. Motor impairment in posture, gait, and balance is common in individuals with PD (Jankovic and Kapadia, 2001; Jankovic, 2008; Weintraub et al., 2008). The potential for the Strength–Dexterity test to provide information about systemic and gross motor control is attested to by the findings, in which dexterity measures tended to be correlated between the fingers and legs of an individual (Lawrence et al., 2014).

Measures of dynamic force control within the Strength– Dexterity test reflect the degree of hand motor impairment in individuals with PD, potentially fulfilling the need for more objective measures of sensorimotor function. Measuring force variability when the motor system is pushed to a limit of performance (as in the Strength–Dexterity test) may represent a valuable strategy in assessing motor control in both health and disease. Our measures appear to be informative of symptom severity in PD; however, further research is required to determine the effects of disease progression and medication level on performance of the Strength–Dexterity test. We also hope to enable future studies of its underlying mechanisms by developing measurements of force variability or other measures of performance during well-defined tasks. Such measures may prove valuable for monitoring changes in motor impairment, determining dosages for medication, appropriate parameters for deep brain stimulation, or even for early detection of PD. What is more, such dynamical tasks may also be used for rehabilitation to improve sensorimotor function in dexterous manipulation in clinical populations by challenging the motor system at the edge of instability.

## Conflict of Interest Statement

Francisco J. Valero-Cuevas holds US Patent No. 6,5337,075 on some of the technology used, but has no active or pending licensing agreements with any commercial entity. None of the other authors have any financial or personal relationships with other people or organizations that could inappropriately influence this work.
